# Advances and Challenges in Intranasal Delivery of Antipsychotic Agents Targeting the Central Nervous System

**DOI:** 10.3389/fphar.2022.865590

**Published:** 2022-03-24

**Authors:** Manisha Pandey, Neha Jain, Jovita Kanoujia, Zahid Hussain, Bapi Gorain

**Affiliations:** ^1^ Department of Pharmaceutical Technology, School of Pharmacy, International Medical University, Kuala Lumpur, Malaysia; ^2^ Department of Pharmaceutics, Amity Institute of Pharmacy, Amity University, Noida, India; ^3^ Amity Institute of Pharmacy, Amity University, Gwalior, India; ^4^ Department of Pharmaceutics and Pharmaceutical Technology, College of Pharmacy, University of Sharjah, Sharjah, United Arab Emirates; ^5^ Department of Pharmaceutical Sciences and Technology, Birla Institute of Technology, Ranchi, India

**Keywords:** antipsychotic agents, blood–brain barrier, intranasal administration, brain transport, nanocarriers, improved efficacy

## Abstract

Treatment of central nervous system (CNS) disorders is challenging using conventional delivery strategies and routes of administration because of the presence of the blood–brain barrier (BBB). This BBB restricts the permeation of most of the therapeutics targeting the brain because of its impervious characteristics. Thus, the challenges of delivering the therapeutic agents across the BBB to the brain overcoming the issue of insufficient entry of neurotherapeutics require immediate attention for recovering from the issues by the use of modern platforms of drug delivery and novel routes of administration. Therefore, the advancement of drug delivery tools and delivering these tools using the intranasal route of drug administration have shown the potential of circumventing the BBB, thereby delivering the therapeutics to the brain at a significant concentration with minimal exposure to systemic circulation. These novel strategies could lead to improved efficacy of antipsychotic agents using several advanced drug delivery tools while delivered *via* the intranasal route. This review emphasized the present challenges of delivering the neurotherapeutics to the brain using conventional routes of administration and overcoming the issues by exploring the intranasal route of drug administration to deliver the therapeutics circumventing the biological barrier of the brain. An overview of different problems with corresponding solutions in administering therapeutics *via* the intranasal route with special emphasis on advanced drug delivery systems targeting to deliver CNS therapeutics has been focused. Furthermore, preclinical and clinical advancements on the delivery of antipsychotics using this intranasal route have also been emphasized.

## Introduction

The brain is an extremely delicate and sensitive neuronal organ of the central nervous system (CNS), which requires a steady supply of blood, oxygen, and nourishment to sustain homeostasis and other key activities. Diseases of the CNS, often known as CNS disorders, are a collection of neurological disorders that impact the anatomy and physiology of the brain or spinal cord, which together make up the CNS (Upadhyay, 2014). The CNS disorders, specifically neurodegenerative disorders, are the prime challenge and necessitate the immense attention of scientists to develop therapeutic strategies against them. Currently, one of the biggest reasons for disability and death in the world includes neurological disorders. In 2019, Feigin et al. deliberated about the contribution of CNS disorders to the global disease burden. In 2016, the biggest cause of disability-adjusted life-years was neurological illnesses [276 million (95 percent UI 247–308)] and also the second most common cause of mortality [90 million (88%–94%)] (Feigin et al., 2016). So far, neurological illnesses such as Alzheimer’s disease (AD), Parkinson’s disease (PD), psychosis, Huntington’s disease (HD), head trauma, brain tumors, and epilepsy are difficult to diagnose and treat because of the impervious nature of the blood–brain barrier (BBB) (Khambhla et al., 2016). Among these neurological disorders, AD progresses slowly and is the leading cause of dementia. According to data, the incidence and prevalence rate are higher among the elderly (Gorain et al., 2020b; Lei et al., 2021).

On the other hand, PD is another most common progressive neurodegenerative condition, characterized by the death of dopaminergic neurons in the substantia nigra of the midbrain and the formation of -synuclein aggregates (Lewy bodies). Currently, it is thought that PD pathology is not only restricted to a single section of the brain but also includes additional brain regions, neurotransmitters such as acetylcholine (Ach) and dopamine imbalances, and protein aggregates other than Lewy bodies (Radhakrishnan and Goyal, 2018). Alternatively, psychosis is a group of conditions that culminate in a distorted perception of reality. It can be a sign of more serious mental illnesses. People suffering from psychosis may have delusions and hallucinations (Schrimpf et al., 2018). Patients with HD-like CNS disorders are associated with loss of neurons in the striatum and other parts of the brain, resulting in increasing motor, cognitive, and psychiatric symptoms. In addition, multiple sclerosis (MS) is another CNS disorder, which is an autoimmune and inflammatory illness that progresses over time. It is a multifaceted disease in which the immune system of the body attacks the neurological system. In addition, brain tumors, viz., glioblastoma multiforme, a malignant glioma, are the most common type of CNS tumors. It can be benign, beginning and remaining within the brain, or metastatic, originating and residing outside the CNS. Epilepsy is a CNS illness defined by an abnormal increase in brain electrical activity that can be localized or widespread throughout the brain, resulting in partial or generalized seizures (Manford, 2017). Stroke is a type of acute CNS illness in which the vasculature supplying the brain is disrupted, resulting in rapid symptoms that can last anywhere from seconds to hours depending on the amount of brain tissue damaged and severity of the stroke (Prabhakaran et al., 2015). Although a large range of prospective medications has been studied to treat a variety of neurological illnesses, their therapeutic success is still restricted due to the presence of different obstacles. The difficulty in transporting medicines, diagnostic agents, theranostics agents, proteins, nucleic acids, and other macromolecules to the CNS has been restricted by the presence of the BBB and blood–cerebrospinal fluid barrier (BCSFB). Among the two, the strategies to penetrate the BBB are one of the most important challenges encountered by formulation scientists (Banks, 2016).

Therefore, the current neurotherapeutics has two key drawbacks: they have restricted entry of therapeutics by the rigid BBB, resulting in insufficient entry into the brain, and they have restricted access to immune cells in the brain. Most conventional drug treatments for neurological diseases are created with lipophilic characteristics; however, the molecular weights of such compounds are more than 400–500 Da. Due to these properties, these conventional therapeutic components are restricted to cross the rigid biological barrier of the brain to deliver therapeutically effective concentration to the brain (Howes and Kaar, 2018). On the other hand, the trans-cellular mechanism plays an important role in the penetration of small lipophilic molecules, viz., alcohol and steroid hormones (Curley and Cady, 2018; Banks and Greig, 2019). Similarly, antipsychotic agents are also known to present numerous complications, although the patients undergo normal homeostasis. There might be some mild issues of dry mouth or sedation to extremely disagreeable situations of sexual dysfunction, extrapyramidal symptoms, or constipation; to stressful situations (such as acute dystonias); to traumatizing conditions, including tardive dyskinesia or weight gain; and to potentially lethal conditions due to agranulocytosis or myocarditis (Muench and Hamer, 2010).

Thus, the challenges of delivering the therapeutic agents across the BBB to the brain require immediate attention for recovering from the issues by the use of modern platforms of drug delivery and novel routes of administration. Therefore, the present review has been attempted to present the challenges of delivering the therapeutics to the brain using conventional routes of administration and overcoming the issues by the introduction of intranasal routes of administration to deliver the therapeutics circumventing the biological barrier of the brain. We have also emphasized different problems with solutions in administering therapeutics using intranasal routes of administration with special emphasis on advanced drug delivery systems to deliver therapeutics to improve psychotic conditions. Finally, clinical advancement in the delivery potential has also been highlighted in overcoming the challenges of delivering psychotics to the brain.

## Challenges Associated With Brain-Targeted Drug Delivery

Conventionally available therapeutics targeting the CNS is available for oral administration, including antipsychotics. Commonly, the oral route of drug administration of medications represents the most comfortable and prevalent form to human patients because of ease of administration, improved patient compliance, less strict sterility standards than sterile parenteral drugs, and reduced costs for both the producer and customer (Reinholz et al., 2018). Although there are various types of oral formulations available, these orally administered medications can reach their target *via* navigating various body compartments, which is difficult for a wide range of pharmaceuticals (Homayun et al., 2019). The stomach’s harsh acidic pH (1.5–4), first-pass metabolism process, and presence of proteases (e.g., pepsin and cathepsin) prevent the delivery of small and macromolecules *via* this oral pathway (Homayun et al., 2019; Üstündağ Okur et al., 2019). Furthermore, once the drug crosses the biological gastrointestinal barrier, the drug molecule needs to cross an even more rigid barrier of the BBB to reach the CNS (Choudhury et al., 2021); the structure and role in preventing penetration to the BBB have been discussed in the latter half of this section. To surmount the problems associated with oral delivery, the parenteral route is considered as an alternate that possesses advantages of immediate onset of action, and poorly absorbed drugs can be absorbed very well *via* this route (Gulati and Gupta, 2011). On the other side, the drawbacks of parenteral deliveries include its high cost, the need to be sterile, possibilities of infections and nerve damage, and requirement of trained staff for drug administration. However, administration of therapeutics to the systemic circulation *via* parenteral routes of drug administration restricts the penetration of therapeutics by the presence of the BBB. Thereby, the failure to attain therapeutically active concentration at the diseased site became a significant constraint, limiting the therapeutic efficacy of several potential candidates for CNS diseases. Developing an effective CNS drug delivery tool in tandem with CNS drug discovery is critical.

### Blood–Brain Barrier in Resisting Permeation of Therapeutics to the Brain

The human brain comprises 100 billion blood capillaries and the most perfused tissue of the body (Van Tellingen et al., 2015). The brain blood vessel vasculature is designed in such a way as to protect the brain from the entrance of neurotoxic substances, however allowing essential nutrients to maintain the primary function of the CNS. This circulation blood barrier of the brain is known as the BBB. BBB signal conductance is maintained by the ionic composition of the brain, although the entry of macromolecules, neurotoxic substances, and unwanted cells is restricted for the safety of body control. Pericytes and astrocytes on endothelial cells ensure the structural integrity of the BBB. Structural rigidity is maintained by pericytes, whereas basal endothelial layers of the blood vessels are covered by astrocytes and connected with the neurons (Abbott et al., 2006; Alvarez et al., 2013; Kesharwani et al., 2016).

**FIGURE 1:**
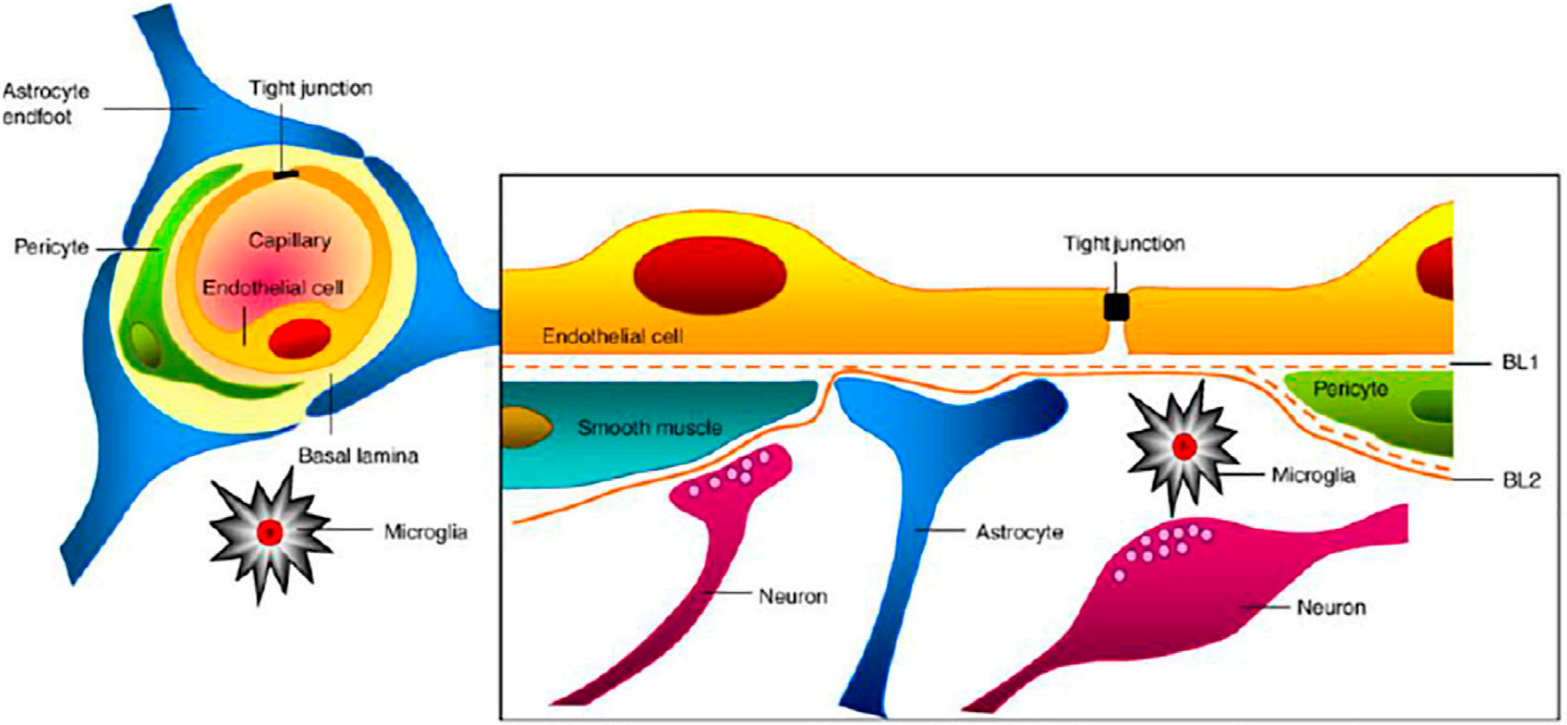
Association of different cells at the blood–brain barrier (Abbott et al., 2010).

Moreover, transmembrane proteins (claudin-1, occludin, claudin-5, etc.) and cytoplasmic proteins (cingunin; zonula occludens-1, 2, and 3; etc.) help in maintaining the closely packed tight junction between brain endothelial cells (Engelhardt and Sorokin, 2009; Amjad et al., 2015) (Figure 1). Only gaseous substances and small molecules are allowed to pass through the BBB. The absence of small apertures inhibits the transendothelial passage of soluble components from the systemic circulation to the brain or vice versa. The lack of any single element may affect the activity and integrity of the BBB. The parallel diffusion of the drugs through the BBB was also restricted by the high electrical resistance of the brain barrier (Butt et al., 1990).

Therefore, the substance, including therapeutic agents, can penetrate the BBB through active or passive diffusion. Active transport requires a carrier protein for transportation, whereas passive diffusion of the drug is concentration gradient–dependent and the most common mode of drug transport. Moreover, permeability depends on some physicochemical parameters, such as molecular weight, lipophilicity, and charge (Mangas-Sanjuan et al., 2010; Carpenter et al., 2014). The drug transport across the BBB by the trans-cellular pathway is constrained by efflux transporter such as P-glycoprotein (P-gp), which expels the drug or substance from the absorbed cell and hinders the penetration in the brain. The majority of drugs used to treat CNS disorders is the potential substrate of P-gp and are unable to achieve an adequate therapeutic concentration at the site of action. Furthermore, the efflux of the drug is also associated with multidrug resistance protein-1 from the plasma membrane of epithelial cells. Hence, smaller lipophilic molecules that could be absorbed from the BBB cannot reach the targeted site due to efflux by multidrug resistance protein-1 and P-gp to the systemic circulation (Ohtsuki and Terasaki, 2007).

The pharmaceutical community’s focus has recently shifted to developing novel and more efficient drug delivery systems, potentially resulting in more effective and safer CNS medicines (Khambhla et al., 2016; Stroup and Gray, 2018)**.** Concurrently, scientists are focusing on establishing alternate routes of drug administration to circumvent the biological barrier of the brain. In this context, the intranasal route of drug administration has shown great potential in delivering therapeutics directly to the brain for its improved efficacy. The connecting section of the manuscript had been focused on this novel route of drug administration with special emphasis on brain delivery.

## Intranasal Routes of Drug Delivery as a Potential Platform to Deliver Therapeutics to the Brain

The role of the BBB toward restrictions in delivering antipsychotic agents to the CNS is limited by the systemic administration of therapeutics. Thus, the treatment of neurological disorders faces a substantial challenge where the endothelial cells of capillaries of the brain and spinal cord restrict the passage of neurotoxic substances (Bonferoni et al., 2019). Alternatively, intranasal administration has been used for various purposes since the last century, including nasal decongestion, rhinitis, and migraine (Erdő et al., 2018).

The mucosa of the nasal cavity provides a great choice of a delivering route for targeting the therapeutics to the brain, where high permeability of the mucosal layer allows penetration of smaller molecules and biopharmaceutical compounds to the CNS (Gänger et al., 2018). Based on its importance in delivering therapeutics to the CNS, recent attention has been drawn toward the intranasal administration of therapeutics, considering an effective alternative to systemic exposure. This novel route of administration provides a large surface area with high vascularity and the possibility to circumvent the BBB where painless delivery of the therapeutic agent is possible with minimal invasion (Gorain et al., 2018; Kim et al., 2018; Gadhave et al., 2019). In addition, this route could avoid all the drawbacks of other routes of administration where rapid drug absorption leads to a rapid onset of action (Jain and Patravale, 2009; Choudhury et al., 2019). The Red Indians of North America have utilized crushed leaves of *Ranunculus acris* to treat headaches by nasal inhalation (Xu et al., 2020) because the administration of low doses using this intranasal route provides therapeutic effects with few systemic adverse effects.

Furthermore, compared to the smaller surface area of the lung, the intranasal administration is considered an effective route for drug delivery, especially for pharmaceuticals with low aqueous solubility (Alshweiat et al., 2019). Since the BBB does not encompass the olfactory area of the nose, evasion of the BBB after intranasal delivery is feasible. The intranasal route offers good patient compliance as no technical skills or expertise is required for the administration of intranasal formulations. As a result of the increased medication bioavailability to the CNS, fewer drug doses are required *via* the intranasal route.

The intranasal route offers a special anatomical feature that enables the transportation of drugs directly to the CNS and systemic circulation (Kashyap and Shukla, 2019). The nasal channel travels from the nasal vestibule to the nasopharynx and is 12–14 cm long. It is divided into three sections: vestibular, respiratory, and olfactory. The nasal septum separates two chambers (i.e., nostrils) with a capacity of 16–19 cm^3^ and a surface area of roughly 180 cm^2^. The vestibular area filters particles from inhaled air and is positioned at the front aperture of the nasal passages (Miyake and Bleier, 2015). In this region, however, drug administration and absorption are of utmost importance. Hair covers this area, filtering inhaled air and preventing airborne contaminants from entering the respiratory system. The respiratory system has a huge surface area and significant vascularity (about 130 cm^2^). Most of the medication absorption takes place in this area. This area is lined with the pseudostratified columnar epithelium and covered with a thick coating of mucus that flows toward the nasal cavity’s posterior openings due to ciliary rhythmic motions. The olfactory region, which has a surface area of around 15 cm^2^, is vital for delivering drugs to the brain and cerebrospinal fluid (Bharadwaj et al., 2021). It comprises thick connective tissue and the lamina propria, which houses the olfactory epithelium. The nasal mucosa thickness varies between 2 and 4 mm. To capture undesirable particles, epithelial cells border the nasal canal and are covered by a 5-m-thick mucus coating (Patel, 2017).

Bypassing the biological barrier (BBB) and avoiding clinically incompatible routes of direct administration of therapeutics to the region of interest in the brain, therapeutics could be targeted to the specific regions of the brain using the nanotechnology-based cargo. Exposing the olfactory region of the nasal cavity with these advanced delivery tools has been shown to improve transportation of the therapeutics to the brain, even the peptides and genetic materials of interest (Baba et al., 2015). The pathway used to transport the genetic material loaded nanocarrier *via* olfactory bulbs has been shown to restore the olfaction of the olfactory deficit condition (Baba et al., 2015). The layout of the CNS provides the stages of delivering the absorbed component from the olfactory bulb to the piriform, entorhinal cortex, amygdala, hippocampus, and ventral tegmental area (Dremencov et al., 2021). With this brain delivery approach, the connecting section has highlighted the underlying mechanisms of transportation of the therapeutics across the nasal epithelium.

### Mechanisms of Drug Transport Across the Nasal Epithelium

**FIGURE 2:**
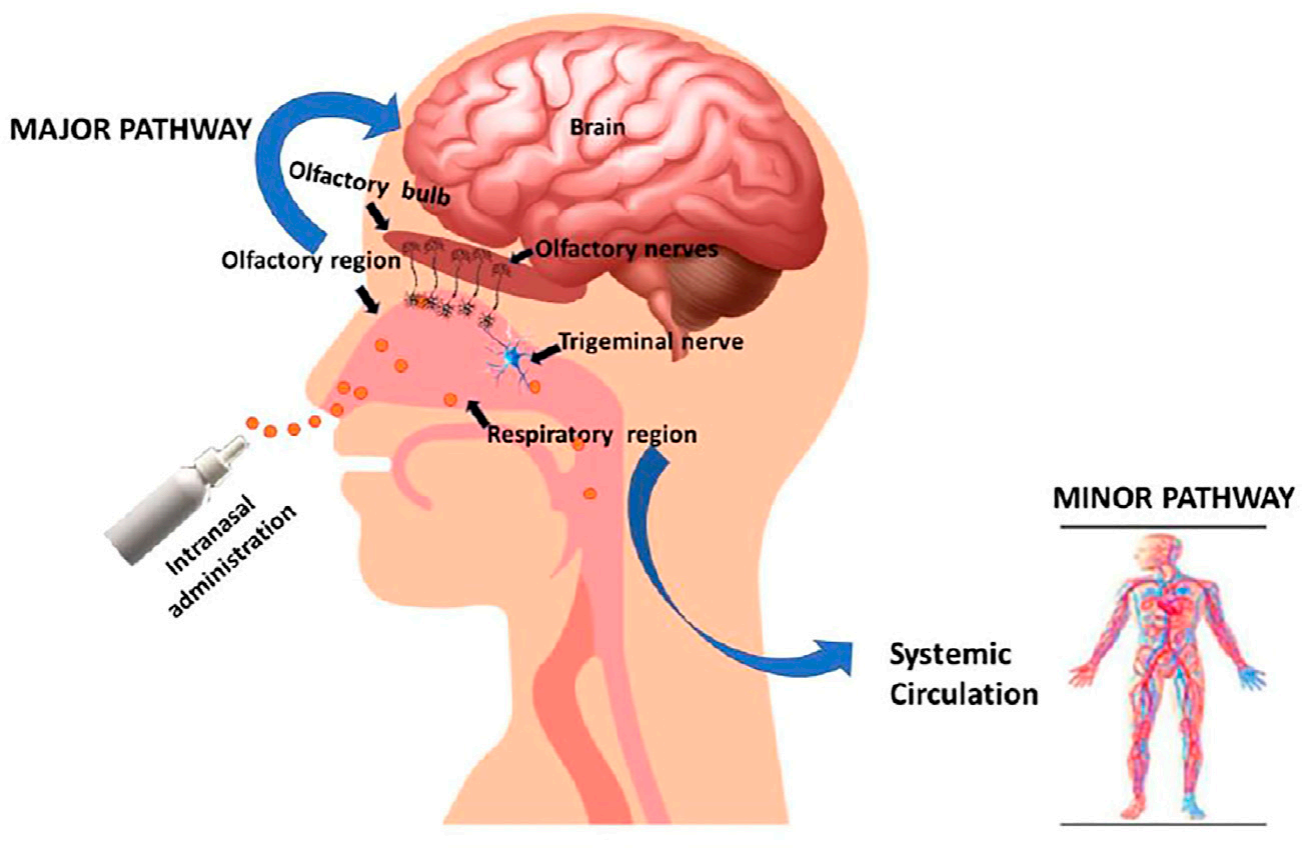
Mechanism of drug delivery *via* the intranasal route of administration.

For facilitation of absorption to occur, the medicine must penetrate through the mucus layer of the nasal cavity, which comprises the respiratory and olfactory epithelium, as shown in Figure 2. The human nasal epithelium is pseudostratified and comprises four different types of cells (goblet, basal, nonciliated, and ciliated columnar microvillus cells). In the turbinates, the most prevalent types of cells are ciliated cells. The olfactory region of the intranasal cavity is not ciliated, whereas the respiratory area (nasal fossa) is ciliated. Columnar (goblet, ciliated, and microvillus) cells are found in the apical side of the cell layer, close to the lumen.

In contrast, basal cells are found in the basolateral side of the epithelium, closer to the basal lamina. The goblet cells produce mucus, which is then cleared by the ciliated cells through mucociliary clearance (Tildy and Rogers, 2015). Charged molecules have a harder time passing through mucus than uncharged molecules. There are two basic pathways for medication absorption *via* the nasal mucosa: trans-cellular and paracellular absorption (Wang et al., 2019). The paracellular pathway is energy-free and involves a slow passive diffusion of the drug through the gaps between the cells. In general, the degree of intranasal absorption reduces with increase in molecular size of the drug (Ozsoy et al., 2009). A medication with a molecular weight greater than 1 kDa, for example, has low bioavailability after intranasal delivery. However, absorption enhancers can improve the bioavailability of these compounds. Transcellular absorption, on the other hand, is applicable to lipophilic medicines that are easily absorbed *via* the epithelial cells of the nose, which may be mediated by carriers or involve the opening of tight junctions for drug absorption. P-gp, transporters such as organic cation, dopamine, and amino acid are all located in the nasal mucosa, particularly in the mucosa of the olfactory region (Wang et al., 2019). The olfactory region is positioned above the middle turbinate and contains smell receptors and millions of olfactory nerve terminals. The olfactory bulb (gray matter) in the brain is the place where the nerve ends. Mucus produced in the olfactory glands (Bowman’s) moistens the olfactory epithelium, which dissolves odorants and aids in olfactory transduction response. Chang et al. evaluated the feasibility of delivering mitochondria intranasally to bypass the BBB in treating PD on the ipsilateral sides of lesioned brains for 3 months. Researchers delivered allogenic mitochondria conjugated with Pep-1 (P-Mito) or unconjugated (Mito) in the form of intranasal infusion once a week. A significant improvement of rotational and locomotor behaviors in PD rats was observed. Investigators revealed that the mitochondria penetrated *via* the accessory olfactory bulb and doublecortin-positive neurons of the rostral migratory stream (RMS) on the ipsilateral sides of lesions. This investigation shows the potential of intranasal delivery of mitochondria validating internalization and migration of the mitochondria *via* RMS neurons in the olfactory bulb (Chang et al., 2021)**.** Furthermore, Tang et al. formulated dopamine-loaded borneol and lactoferrin co-modified nanoparticles (Lf-BNPs) using a double emulsion solvent evaporation method to obtain high therapeutic efficacy and reduced side effects. Investigators have performed *in vitro* cytotoxicity studies in SH-SY5Y and 16HBE cells and *in vivo* pharmacokinetic studies on rat brains. The results revealed the effectiveness of dopamine Lf-BNPs administered intranasally for the management of PDs (Tang et al., 2019). Finally, endocytosis is the mechanism through which drugs are transported, particularly by swallowing into the cell. The M cells are responsible for particle uptake by the nasal epithelium. Endocytosis is the most common mode of transport for molecules with molecular weights greater than 1,000 Da, such as proteins, peptides, polypeptides, and polypeptide-coated nanospheres in the 500-nm range (Wang et al., 2019). Even though this route has been explored by several scientists around the world for delivering therapeutics in the brain, there are a number of limitations that restrict transformation into clinical application. Those challenges are demonstrated in the subsequent section of the article for better insights into this delivery route.

## Challenges of Delivering Therapeutics *via* the Intranasal Route

Drug delivery *via* the intranasal route has a plethora of benefits, as discussed in the earlier section. Still, several factors, viz., biological, biopharmaceutics, and physicochemical properties of drug molecules require special attention while formulating an intranasal formulation. Despite special anatomical features for intranasal drug absorption, the therapeutic target areas for nasally administered medications differ depending on whether they are meant to have a local, systemic, or presumably CNS effect. This means that the medicine must be deposited in certain locations of the turbinate and olfactory regions for these goals. This is not a simple process to be accomplished precisely (Schroeter et al., 2006)**.** The complicated features and complex geometry of the human nasal chamber, notably the difficult-to-reach sinuses, make medication deposition and subsequent absorption difficult (Liu et al., 2009). Inhaled drug particles are trapped in the anterior region due to the convoluted nature of the airway and only reach the olfactory region insignificantly. Biologically, this route of drug delivery is not suitable in various situations. During pathological conditions such as nasal congestion in rhinitis and sinusitis, absorption of active is adversely affected, and interpatient variabilities can also be noted for absorption of the same drug.

In comparison to the gastrointestinal tract, the intranasal route has a small surface area for drug absorption. Moreover, defense mechanisms associated with the intranasal route, viz., mucociliary clearance can affect the drug absorption, and enzymes present in the nasal cavity may also degrade administered drugs. Furthermore, high molecular weight drugs cannot be administered and the volume which can be delivered intranasally is limited to a very low dose, that is, 25–200 µL (Miyake and Bleier, 2015). When developing an effective and safe pharmacological formulation for intranasal administration, safety is paramount. During the development phase, the safety of the drug and the excipients in the formulation must be considered. Large molecules, such as peptides and proteins, require absorption enhancers. They improve the permeability of the nasal mucosa, which increases medication bioavailability after intranasal delivery (Davis and Illum, 2003). Other excipients serve as mucoadhesives, extending the period in contact with the nasal mucosa. Excipients can dramatically reduce the safety of the final medication due to their own toxicity profile and the drug’s enhanced local exposure time. The toxicological implications of the drug formulation’s local, systemic, CNS, and pulmonary effects must also be examined. First, let us discuss all the biological factors which present challenges for intranasal drug delivery such as blood circulation to the nasal mucosa, presence of protective enzymes, and nasal defense mechanism, that is, mucociliary clearance (Miyake and Bleier, 2015). Blood circulation to the nasal area controls crucial nasal parameters such as temperature and humidification of inhaled air. Available vasoconstrictors, the most popular decongestants, viz., xylometazoline and oxymetazoline, were found to reduce blood flow within the nose. Nasal hemorrhage and the extremely unusual occurrence of nasoseptal perforation were reported as rare adverse effects with intranasal corticosteroids used for treating seasonal rhinitis (Bende et al., 1992). Furthermore, oxymetazoline, an anesthetic for the treatment of epistaxis, has its strong peripheral alpha-adrenergic 1 and 2 agonists, but it can also activate central alpha 2 adrenoreceptors when it reaches the systemic blood circulation. Vasoconstriction and sympathetic effects, such as fast, irregular, or pounding heartbeat, headache, dizziness, sleepiness, elevated blood pressure, nervousness, and trembling, are examples of undesirable systemic effects caused by oxymetazoline which may worsen hypertension, tachycardia, and peripheral vasoconstriction (Dokuyucu et al., 2015). These challenges need to be addressed before addressing any CNS issues using the intranasal route. The subsequent section of the article highlighted recent advancements in the delivery of antipsychotics by overcoming the associated challenges in intranasal delivery.

## Recent Advancement of Delivering Antipsychotics Using Intranasal Route

**FIGURE 3:**
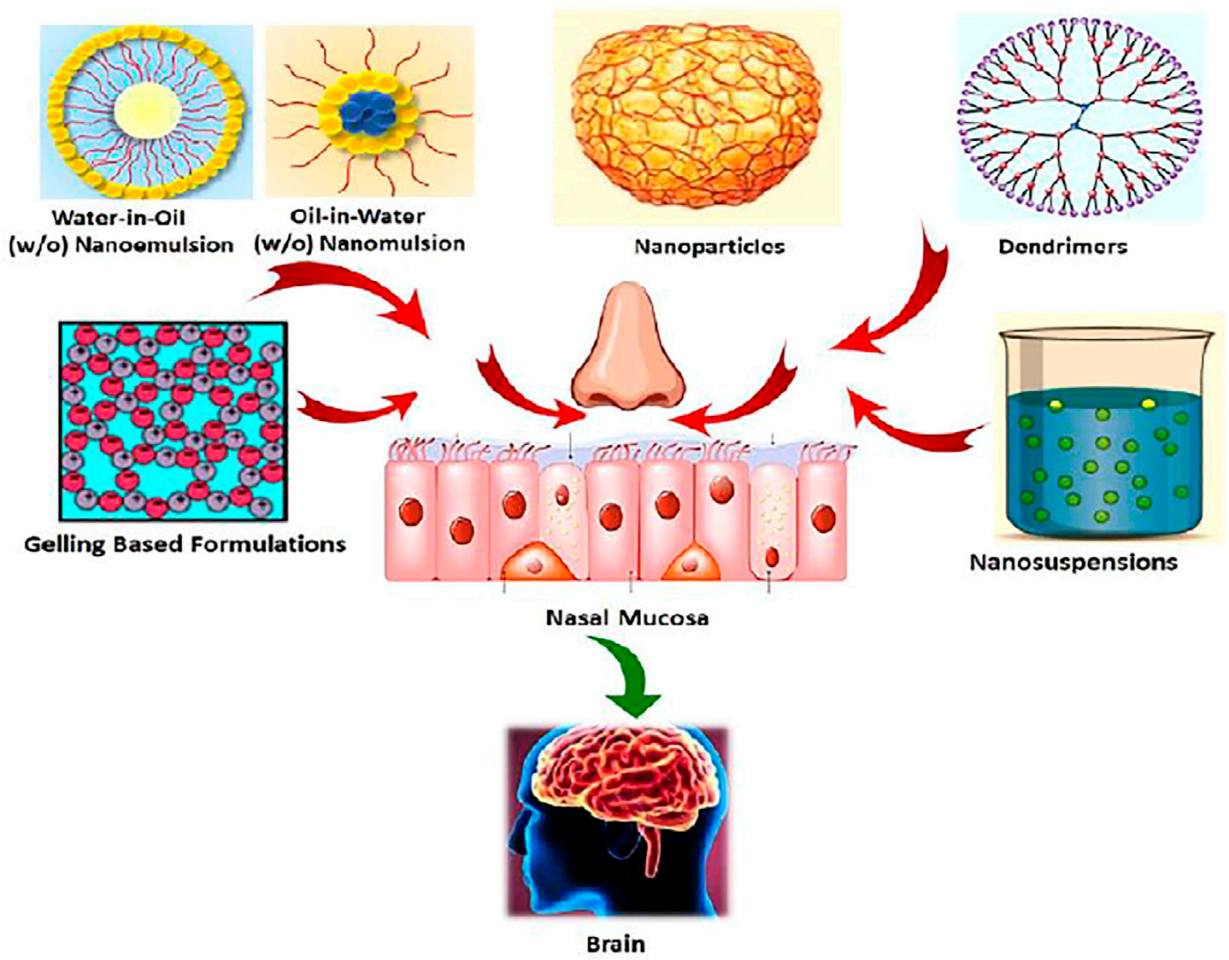
Various advanced drug delivery tools for nose-to-brain targeting of neurotherapeutics.

In recent years, the delivery of antipsychotic agents through the intranasal route has been extensively investigated to unravel various challenges over the other conventional routes. Among the wide range of delivery systems, some expanded specific attention in the drug delivery process. The last 5 years advocate the latest advancement in intranasal delivery of antipsychotic drugs through various delivery systems, including nanoemulsions, lipid nanoparticles (SLNs and NLCs), nanosuspensions, dendrimers, and *in situ* gels as illustrated in Figure 3. Various antipsychotic drugs such as aripiprazole, asenapine, olanzapine, zotepine, clozapine, amisulpride, paliperidone, quetiapine, and haloperidol were explored for intranasal administration through advanced delivery systems and have been reported in the literature (Supplementary Table S1).

### Nanoemulsion Tool in Intranasal Delivery of Antipsychotics

Nanoemulsion is a dispersion of two immiscible liquids (usually water and oil) in the form of droplets ranging from 20–200 nm. This dispersion system can efficiently deliver the lipophilic therapeutics across the BBB due to high physical stability, biodegradability, nano-range droplets, and biocompatibility (Pandey et al., 2018; Chatterjee et al., 2019). In this regard, aripiprazole-loaded mucoadhesive nanoemulsion had been fabricated and investigated by Kumbhar et al. Aripiprazole is currently approved for the treatment of schizophrenia against both negative and positive symptoms with a safer profile than other categories of drugs. However, its poor solubility, bioavailability, and nontargeted delivery result in different side effects, losing patient compliance when delivered *via* the conventional oral route of administration. Therefore, the researchers hypothesized that mucoadhesive nanoemulsion could entrap the drug in the oil core of the formulation, and nasal delivery can enhance its bioavailability through longer retention of the formulation due to the presence of mucoadhesive polymers in it. The nanoemulsion was found efficient for intranasal delivery with 96.9% targeting efficiency of the drug. The nanoemulsion was optimized using the Box–Behnken statistical design and able to permeate well (permeation co-efficient: 62.87 cm h^−1^ × 10^3^) through the mucous membranes of sheep. Maximum Cmax values and Tmax value of aripiprazole after intranasal administration of the nanoemulsion were found to be 15.19 ± 2.51 μg ml^−1^ and 1 h, respectively. The report also discussed that components and formulations are less toxic as per the *ex vivo* ciliotoxicity study on nasal mucosa (Kumbhar et al., 2021).

**FIGURE 4:**
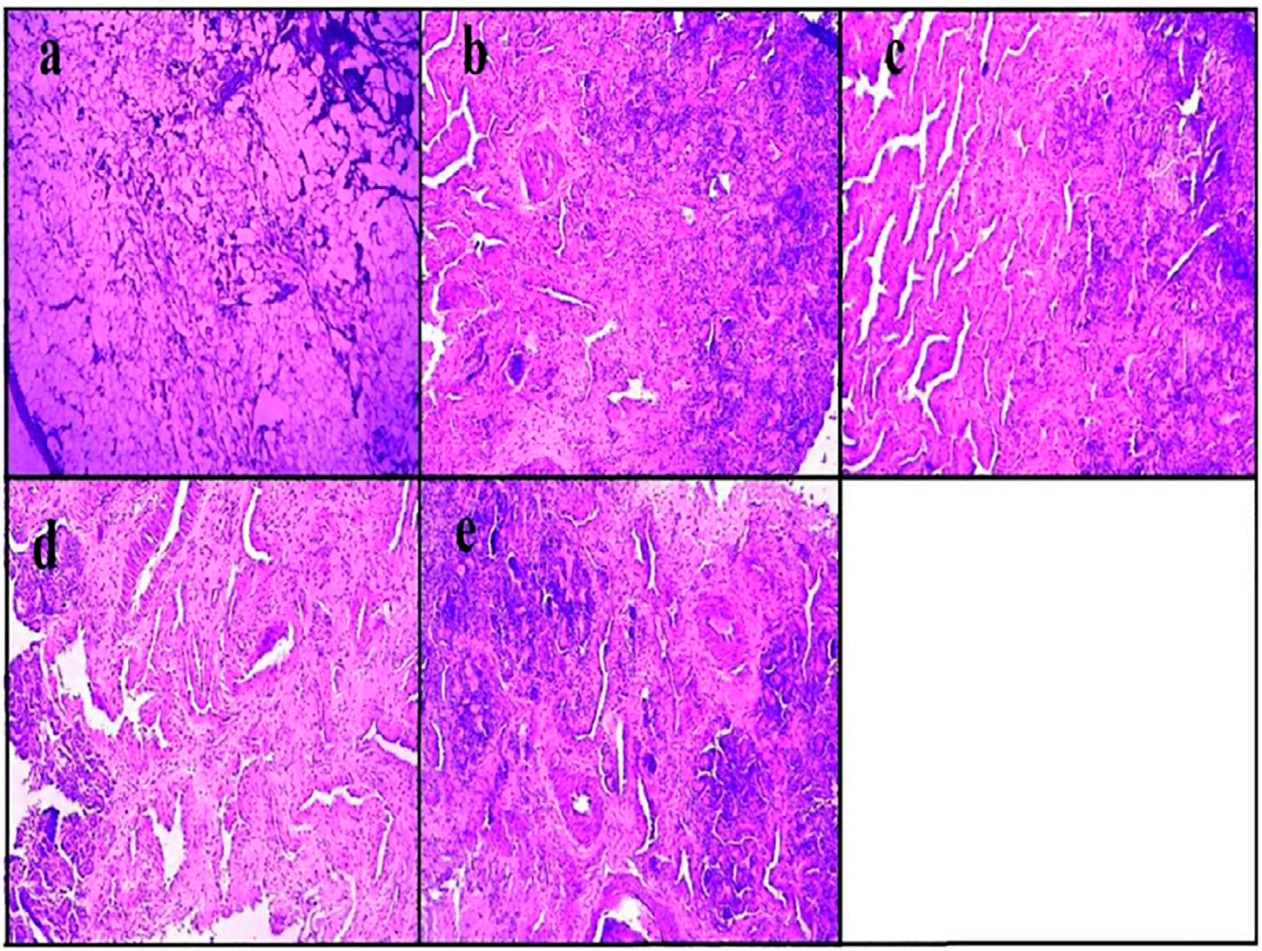
Histopathological assessment of nasal mucosa condition after 2 h exposure of **(A)** iso-propyl alcohol (positive control), **(B)** simulated nasal fluid (pH 6.4) (negative control), **(C)** asenapine mucoadhesive nanoemulsion, **(D)** asenapine nanoemulsion, and **(E)** blank mucoadhesive nanoemulsion (Kumbhar et al., 2020).

**FIGURE 5:**
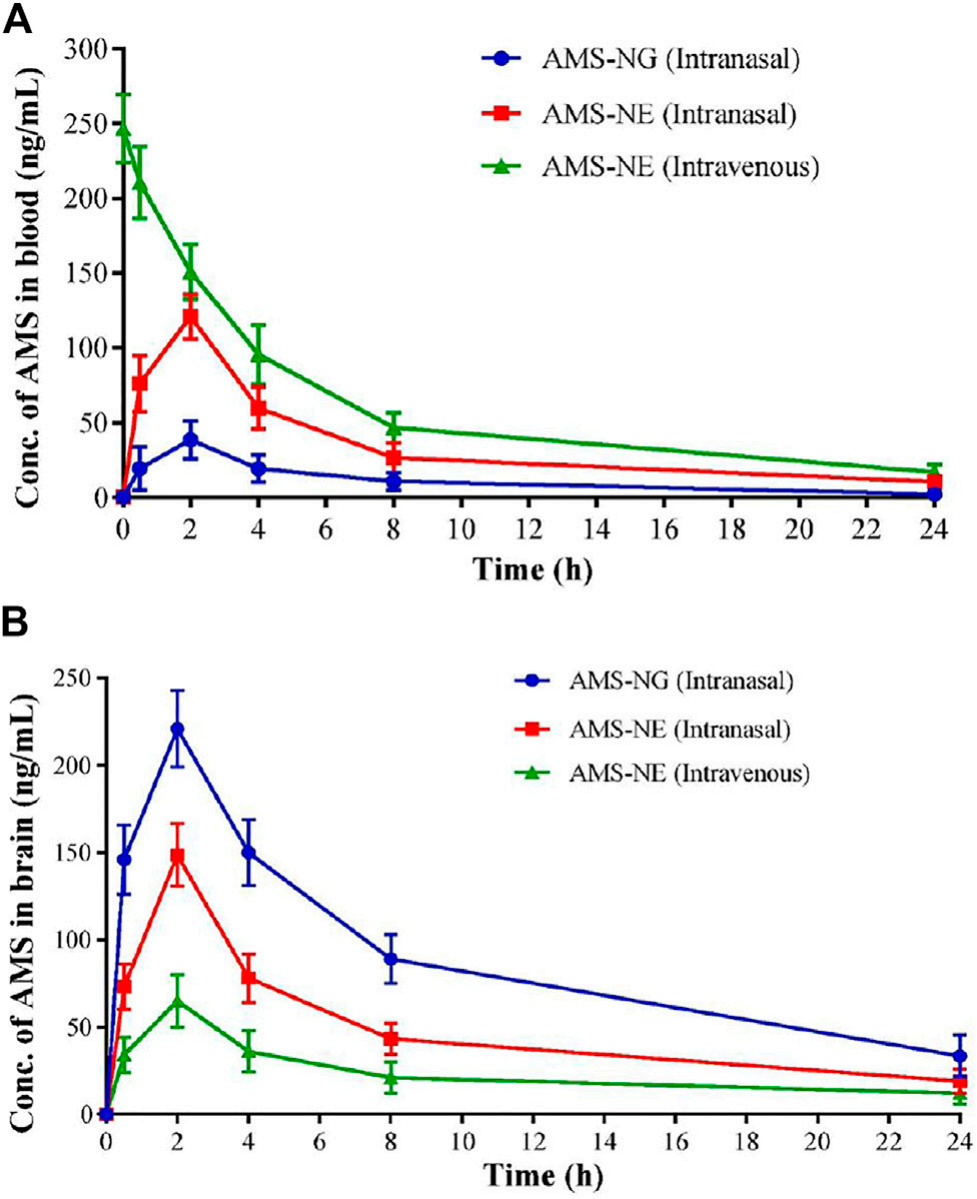
Mean **(A)** blood, **(B)** brain concentration-time curve for amisulpride nanogel (AMS-NG) (intranasal), amisulpride nanoemulsion (AMS-NE) (intranasal), and AMS-NE (intravenous) (Gadhave et al., 2021).

Similarly, asenapine maleate, approved by the Food and Drug Administration for the treatment of schizophrenia and bipolar disorder, belongs to the BCS class II category with limited aqueous solubility with extensive first-pass metabolism, which leads to low bioavailability (<2%). Thus, the same investigators explored the intranasal route for administering asenapine-loaded mucoadhesive nanoemulsion (ASP-MNE) to investigate the drug targeting potential. Droplet size distribution, droplet size, and surface charge of formulated nanoemulsions were optimized using a statistical design, Box–Behnken design. The optimized formulation showed a droplet size of 21.2 ± 0.15 nm with a spherical shape and acceptable polydispersity index (0.355). The results showed enhanced *ex vivo* permeation with ASP-MNE and asenapine nanoemulsion (ASP-NE) when compared to the drug solution. Toxic manifestation on the nasal mucosa by the use of excipients and formulation was evaluated *via* the nasal ciliotoxicity study of sheep nasal mucosa, where the mucosa treated with simulated nasal fluid did not show any histological damage; in contrast, positive control (*iso*-propyl alcohol) leads to extensive damage (Figure 4). It is also evident from the figure that blank formulation, ASP-NE, and ASP-MNE did not show any morphological changes or toxicity to the nasal mucosa. The pharmacokinetics data revealed that intranasal delivery of asenapine in animals increases the concentration of drug (284.33 ± 5.5 ng/ml) in the brain. The formulations were also found to be safe with better antipsychotic activity through hind-limb retraction test and good locomotor activity (Kumbhar et al., 2020). In another study, Gadhave et al. fabricated and evaluated amisulpride-loaded *in situ* nanoemulgel for intranasal delivery. The researchers first developed, optimized, and characterized amisulpride-loaded nanoemulsions. The optimized nanoemulsion was then converted into an *in situ* nanoemulgel using gellan gum and poloxamer 407. Figure 5 indicates that the administration of intranasal amisulpride nanoemulgel and nanoemulsion could lead to significant increase in brain concentration as compared to the brain kinetics following intravenous administration. Moreover, on evaluation of the pharmacokinetic profile, Cmax of the intranasal amisulpride-loaded *in situ* nanoemulgel was found to be 1.48 times higher than that of intranasal amisulpride-loaded nanoemulsion, which indicated that the conversion of nanoemulsion to nanoemulgel facilitated intranasal retention for prolonged absorption (Gadhave et al., 2021). The importance and application of the gel in the delivery of therapeutics *via* the intranasal route have been discussed in the subsequent section.

Overall, the experimental results of nanoemulsion-based intranasal delivery of antipsychotics illustrated superior control of the diseased condition safely, which could open up avenues in the future for further experimentation toward clinical application.

### Nanoparticles in the Advancement of Intranasal Delivery of Antipsychotics

Lipid nanoparticles are nano-based lipid structures capable of encapsulating therapeutics of different characteristics. Lipid nanoparticles can be categorized as NLCs (nanostructured lipid carriers), liposomes, and SLNs (solid lipid nanoparticles) with the potential of loading both hydrophilic and hydrophobic drugs. Olanzapine is one of the most widely used drugs to treat schizophrenia in controlling negative and positive symptoms. The main constraint in olanzapine delivery is the high volume of distribution; hence, only a small percentage of the drug reaches the brain. Thus, cardiovascular side effects and dose-dependent weight gain are the main setbacks of the nonselective distribution of olanzapine. Research study on the development of olanzapine-loaded nanoparticles by emulsification followed by the ultra-sonication technique to reduce its off-target effects is required. The formulation parameters were optimized by the Box–Behnken design, and the optimized formulation was then coated with Polysorbate 80 to increase the brain targeting potential. Formulated SLNs were spherical in shape with a small particle size (151.29 ± 3.36 nm) and narrow size distribution. Extended release of the drug was observed for 48 h, and no significant changes in the formulation parameter after 6 months of the stability study were reported. The olanzapine-loaded SLNs resulted in an enhanced antipsychotic effect (48 h) when compared with pure olanzapine solution (8 h) (Joseph et al., 2017). Similarly, Kaur et al. prepared SLN comprising solid lipid (steric acid), solvent (isopropyl alcohol), liquid lipid (oleic acid), surfactant (Poloxamer 407), and coating agent (chitosan) for olanzapine delivery. They found that NLCs containing olanzapine following administration *via* the intranasal route resulted in a high permeation rate of 84.03%. Entrapment efficiency, particle size, and mucoadhesion study was 87%, 227.0 ± 6.3 nm, and 15 ± 2 min, respectively (Kaur et al., 2021). In another interesting study, olanzapine was investigated to overcome the problem of poor bioavailability and liver metabolism by delivering through chitosan-modified niosomes. The prepared niosomes were evaluated for particle size and entrapment efficiency; moreover, they have reported 2.46 times higher permeation through nasal mucosa (Khallaf et al., 2020).

On the other hand, Jazuli et al. prepared NLC-encapsulated lurasidone for brain delivery *via* the intranasal route. NLC formulation was prepared by the solvent evaporation method and optimized by the statistical design entitled Box–Behnken design for the study of surfactant, sonication time, and lipid concentration. Results of the study demonstrated that the concentration of the drug was found to increase by 2 folds in the brain when compared with the drug solution after intranasal administration (Jazuli et al., 2019). In another study, Upadhyay et al. utilized two strategies in a single platform for the preparation of quetiapine inclusion and then loaded it into the liposomal carrier. Liposomes were prepared by thin-film hydration–sonication and optimized by the 3^2^ full factorial design. The developed liposomes revealed better permeation using the nasal mucosa of sheep with an acceptable safety profile (Upadhyay et al., 2017).

Alternatively, groups of authors reported donepezil-loaded SLNs for delivery of drugs to the brain through the intranasal route. In this study, glyceryl monostearate was used as the solid lipid and Tween 80 and Poloxamer 188 were used as surfactants to prepare SLNs by solvent emulsification diffusion techniques. As per the data of pharmacokinetic and biodistribution studies, drug-loaded SLNs showed that the AUC value was 2.26-fold higher in the brain than in the drug solution after intranasal administration (Yasir et al., 2017). In another interesting report, it was illustrated that hybrid nanoparticles made up of Brij98 (surfactant) and Eudragit L100-55 were able to efficiently deliver the antipsychotic drug, haloperidol. The prepared nanoparticles showed good stability and drug loading efficiency (Filippov et al., 2021).

Alternatively, polymeric micelles are the nanosized, self-assembled, core-shell structures characterized by amphiphilic block copolymers. These micellar structures are capable of loading, carrying, and releasing the drug molecules at a particular targeted site. Interesting features of polymeric micelles, such as high stability, core-shell arrangement, micellar association, low toxicity, biocompatibility, and nano-size (Gorain et al., 2020a; Alfaifi et al., 2020) make it a favorable device for intranasal delivery of antipsychotic drugs. In this regard, Pokharkar et al. attempted to investigate the brain targeting potential of lurasidone-loaded mixed micelles (LHMMs) *via* the intranasal route. The particle size and entrapment efficiency of optimized LHMMs were 175 nm and 97.8%, respectively. The LHMM showed 81% drug diffusion *in vitro* with 79% of drug penetration through the nasal mucosa without any toxicity or morphological changes on mucosal cells. The pharmacokinetic results revealed an enhanced concentration of drug (9.5 ± 0.21 μg/ml C_max_) in the brain after intranasal administration with respect to the pure drug (Pokharkar et al., 2020). Thus, these lipid-based formulations, including SLN, NLC, liposome, micelle, etc. are found to be a useful delivery system for intranasal administration of the antipsychotic drugs for targeting direct brain delivery.

### Nanosuspension-Based Improved Delivery of Antipsychotics

Nanosuspensions are biphasic dispersion systems comprising a drug (particle size less than 1 μm in size) suspended in an aqueous medium (Shariare et al., 2019). Nanosuspension is a very effective strategy for enhancing the solubility and bioavailability of drugs prepared by a variety of methods, such as melt-emulsification, precipitation, microemulsion, milling methods, and high-pressure homogenization (Patel and Agrawal, 2011). Utilizing this novel platform, Pailla et al. reported the formulation development of zotepine-loaded nanosuspension with two methods, high-pressure homogenization preceded by precipitation and sonoprecipitation. Usually, zotepine is a well-tolerated antipsychotic drug with low bioavailability and nonselective distribution, which led to a lower concentration of the drug. To combat this setback, a nanosuspension, comprising hydroxypropyl methylcellulose E15, Pluronic F-127, and soya lecithin, was synthesized. The results showed an average particle size of less than 520 nm with 81.79% of drug release. The nanoemulsion prepared by sonoprecipitation resulted in lower brain concentration (AUC, 8.6-folds) when compared to the homogenization process (AUC, 10.79-folds). However, no significant changes were observed in brain histopathology results (Pailla et al., 2019). Although much exploration has not been made in delivering therapeutics under the antipsychotic category using this nanosuspension platform, this delivery system has the potential to improve the brain availability of these therapeutic agents.

### Advancement of Dendrimer-Based Delivery of Antipsychotics

Dendrimers are branched, discrete structures with a controlled shape, size, molecular weight, and high level of surface functionality (Gorain et al., 2017, 2018). In the last few years, dendrimers were also studied for delivery of drugs through the intranasal route to target the brain bypassing the liver metabolism of the incorporated drug. In this context, an interesting study was carried out by Katare et al. on the formulation and distribution profile of dendrimers consisting of haloperidol. The developed formulation was intended for intranasal administration. The study supported an increase in solubility (more than 100 times) in the developed dendrimer-based formulation and significantly high distribution in the brain. Moreover, similar behavioral responses were observed on administration of 6.7 times less dose of dendrimer–haloperidol through the intranasal route compared to intraperitoneal injection of the same drug. This indicated the potential of dendrimers in brain delivery of hydrophobic drugs (Katare et al., 2015). In another strategy, Xie et al. emphasized using a PAMAM-based-dendrimer-incorporated *in situ* gel for transportation of paeonol for brain targeting after the intranasal administration. The various parameters such as particle size, entrapment efficiency, zeta potential and drug loading for prepared dendrimers and viscosity and mucoadhesive strength for dendrimer-loaded *in situ* gels were characterized. Following intranasal delivery of optimized formulation, maximum concentration was attained at 12 h with significant accumulation of dendrimers at the target area. It was also observed that intranasal brain transportation efficiency was improved after entrapping dendrimers in gel (Xie et al., 2019). Thus, the dendrimer-based formulation platform provides the potential of transporting drugs directly and efficiently to the brain.

### Gelling-Based Delivery Platform of Antipsychotics

Gels are semisolid dosage forms with three-dimensional polymeric matrices comprising a small amount of solids carrying a large amount of liquid (Pandey et al., 2021). The *in situ* gelling system has the capability to convert from sol-to-gel upon application to the site of administration. These systems are easily removable from the skin, have high water content, and are less greasy (Halligan et al., 2017). Poloxamers, gellan gum, carbopol (anionic polymer), chitosan (cationic polymer), alginates, hydroxypropyl methylcellulose, methylcellulose, etc. are the commonly used polymers for the preparation of gelling systems (Cassano et al., 2021). Interestingly, Michael et al. described the preparation of peptide-loaded oxidized starch nanoparticles and carboxymethyl chitosan hydrogel. This novel strategy was capable of delivering drugs from the nose to the brain by evading various barriers. This strategy presents the potential to significantly reduce the dose frequency and side effects of antipsychotics through a minimally invasive route of administration. This strategy offers alleviation of schizophrenic negative symptoms up to 72 h in the schizophrenia rat model at lower doses than intraperitoneal administration of the drug (twice) to achieve therapeutic response for only a few hours (Majcher et al., 2021). Another similar study emphasized the development of clozapine-loaded nanoemulsion based on an *in situ* gel for bioavailability enhancement *via* intranasal administration. Tween 80, Transcutol P, and peppermint oil were selected as components to fabricate the nanoemulsion, which was then characterized for globule size, *in vitro* release, transmission electron microscopy, and viscosity. The developed nanoemulsion was then converted into an *in situ* gel by using Pluronic® F-127 (PF127) and F-68 (PF68). Ciliotoxicity examinations also showed no damage to the epithelium lining and cells of the nasal mucosa after application of the formulation (Abdulla et al., 2021).

The potential of brain targeting through the intranasal route can also be explored by another new delivery system such as mesocellular silica form, hybrid nanoparticles, and cyclodextrin-based inclusions incorporated in *in situ* gels. This study developed an intranasal thiolated chitosan-coated paliperidone-loaded microsphere adsorbed on the mesocellular silica form. The use of thiolated chitosan was found to be effective in enhancing the mucoadhesive strength of the microspheres. The research group was able to successfully control the release of the drug by avoiding the initial burst release (Nanaki et al., 2017). A similar study reported by Sherje and Londhe showed improved permeation of paliperidone through sheep nasal mucosa using a hydroxypropyl-β-cyclodextrin–based paliperidone inclusion platform. In this study, Carbopol 934 and hydroxypropyl methylcellulose K4M were used to formulate the *in situ* gel and found to show sustained drug release with high mucoadhesion strength (Sherje and Londhe, 2018). Overall, the outcomes of the studies demonstrated the superiority of delivering therapeutics to the brain *via* the intranasal route using the *in situ* gel–based platforms.

## Clinical Studies of Treating Psychotic Disorder Using Intranasal Routes

Clinical studies are the next step to evaluate the effectiveness and safety of new delivery devices following formulation optimization using an alternate route based on the data on preclinical studies to send the novel ideas to the bedside of the patients. Research progress using intranasal administration of therapeutics has also been advanced at different stages of clinical research. In this regard, it is pertinent to mention that oxytocin nasal spray combined with social cognition training (SCT) has undergone clinical trials to investigate its efficacy in early psychosis. Early psychosis contributes to poor functional outcomes; however, until now, the search for effective treatment for this problem is still in the pipeline. The suggested treatment efficacy was evaluated for clinical symptoms that improve social cognition and functioning in early psychosis. Fifty-two individuals with the early psychosis schizophrenia spectrum were recruited for a randomized, double-blind, placebo-controlled clinical trial. Participants were administered with placebo or nasal oxytocin spray two times a day for 6 weeks along with SCT. Efficacy was assessed by the Social Functioning Scale, reading the Mind in the Eyes Test, observing positive and negative symptoms. The results concluded that oxytocin treatment could have no benefits on primary and secondary outcomes of early psychosis. However, a further clinical investigation is needed for nasal spray delivery and dose-related variables (Cacciotti-Saija et al., 2015).

Similarly, the researchers also explored intranasal administration of oxytocin for a new therapeutic strategy, in the improvement of autonomic nervous system (ANS) regulation. They have recruited 17 healthy men and 30 men with clinical high risk for psychosis (CHR-P) for randomized, double-blinded, placebo-controlled, crossover magnetic resonance imaging studies. Intranasal placebo or intranasal oxytocin was self-administered by all participants. Subsequently, after 1 h of dosing, high-frequency heart rate (HR) variability (HF-HRV) and estimated HR were recorded. The results indicated that the placebo did not show any significant difference in healthy men and CHR-P men HR or HF-HRV. However, intranasal oxytocin increased HF-HRV in CHR-P only. These findings support the need for further research to investigate the therapeutic potential of intranasal oxytocin (Martins et al., 2020). Hippocampal dysfunction (HD) was investigated as a crucial factor for psychosis onset. Alteration in hippocampal blood flow was observed in CHR-P. In this context, Davies et al. examined the intranasal oxytocin effect on hippocampal blood flow *via* a randomized, placebo-controlled, double-blind study on 30 CHR-P individuals. Intranasal administration of oxytocin was found to enhance the hippocampal blood flow, although merits were suggested to investigate further with more patients (Davies et al., 2019).

In another clinical trial, a correlation between the change in clinical symptoms and peripheral oxytocin levels in schizophrenia patients was investigated. Twenty-eight patients were recruited in 3 weeks of study, and outcomes revealed an improvement of negative symptoms in a small group of patients (Lee et al., 2016; Katare et al., 2017). On the other hand, researchers investigated the efficacy of nasal spray of esketamine for treatment-resistant depression (TRD). Oral antidepressant with esketamine nasal spray was compared with placebo nasal spray with an oral antidepressant for delaying the relapse of depressive symptoms in TRD patients. In this multicenter, randomized, double-blind, clinical trial, 705 adults with TRD were recruited. The outcomes of the study indicated the continuation of the nasal spray of esketamine with an oral antidepressant in patients with TRD showed clinically significant delaying relapse in comparison with the placebo spray with an antidepressant (Daly et al., 2019). Overall, it could be said that the preclinical research toward improvement of brain delivery of antipsychotics would be progressed one step ahead soon for clinical application *via* the effective and safe outcomes in clinical research.

## Conclusion

The rigid biological barrier of the brain (BBB and blood–cerebrospinal barrier) restricts xenobiotics, including therapeutic agents, to enter the brain. Thus, exploring novel strategies of formulation aspects with an alternate route of administration has shown to have a potential impact on brain targeting. Intranasal delivery of therapeutics is proposed to utilize the olfactory and trigeminal pathways to overcome the obstacles imposed by the BBB. Furthermore, this noninvasive route also attracts researchers’ attention, where advanced drug deliveries have shown attractive results in preclinical research. The potential of using novel delivery devices with the polymeric matrix has indicated overcoming the nasomucociliary clearance, thereby allowing prolonged retention of the formulation for constant delivery of therapeutics to the brain. These outcomes are waiting for sufficient safety and efficacy data from the preclinical studies to plan for clinical advancement. The pathways of delivering therapeutics using this intranasal route are yet to be confirmed for reproducible results; thus, this route of drug administration for targeting antipsychotic agents directly to the brain requires future preclinical and clinical investigations.

## Supplementary Material

Supplementary Table S1 Administration of drugs to target the central nervous system through administration of therapeutic deliveries *via* the intranasal route.

## Abbreviations

ASP-MNEASP-MNE, asenapine-loaded mucoadhesive nanoemulsion; AD, Alzheimer’s disease; BBB, blood–brain barrier; BCSFB, blood–cerebrospinal fluid barrier; CNS, central nervous system; HD, Huntington’s disease; LHMM, lurasidone-loaded mixed micelles; MS, multiple sclerosis; NLC, nanostructured lipid carriers; PD, Parkinson’s disease; P-gp, P-glycoprotein; SLN, solid lipid nanoparticle; SCT, social cognition training.
