# Supplementary material for: Advances and Challenges in Intranasal Delivery of Antipsychotic Agents Targeting the Central Nervous System
Source: Front Pharmacol. 2022 Mar 24;13:865590. doi: 10.3389/fphar.2022.865590 (PMC8988043; doi:10.3389/fphar.2022.865590)
Supplement: Supplementary file 1 [file Table1.pdf]

Table 1: Administration of drugs to target central nervous system through administration of thepeutic deliveries via intranasal route

S.No.	Drug	Indications	Aim/ Hypothesis	Research Methodology	Results	Inference	References
**1**	Orexin-A (Hypocretin-1)	age-related cognitive disorders	To investigate the ability of intranasal orexin-A (OxA) administration to improve the anatomical, neurochemical, and behavioural substrates of age-related cognitive dysfunction.	Intranasal OxA administration in aged rats is combined with immunohistochemistry, *in vivo* microdialysis, and attentional-set shifting to assess the effects of intranasal OxA administration on the anatomical, neurochemical, and behavioral deficits that encompass age-related cognitive decline.	Intranasal OxA increases c-Fos expression in several telencephalic brain regions that mediate important cognitive functions, increases prefrontal cortical acetylcholine efflux, and alters set-shifting-mediated attentional function in rats.	these studies provide a framework for the possible mechanisms and therapeutic potential of intranasal OxA in treating age-related cognitive dysfunction.	(Calva et al., 2020)
**2**	Dantrolene	Alzheimer's disease (AD)	To compare the effectiveness and safety of intranasal versus subcutaneous administration of dantrolene in 5XFAD AD mice.	5XFAD and wild type (WT) B6SJLF1/J mice were treated with intranasal or subcutaneous dantrolene (5 mg/kg, 3×/wk), or vehicle. Behavior was assessed for olfaction (buried food test),	Intranasal dantrolene achieved higher brain and lower plasma concentrations than subcutaneous administration. Dantrolene administration at both approaches	The long-term intranasal administration of dantrolene had therapeutic effects on memory compared to the subcutaneous approach even	(Shi et al., 2020)

				motor function (rotarod), and cognition (fear conditioning, Morris water maze). Liver histology (H & E staining) and function, synaptic proteins, and brain amyloid immunohistochemistry were examined. Plasma and brain dantrolene concentrations were determined in a separate cohort after intranasal or subcutaneous administration.	significantly improved hippocampal-dependent and -independent memory in the ETG, whereas only intranasal dantrolene improved cognition in the LTG.	started after onset of AD symptoms, suggesting use as a disease-modifying drug.	
**3**	Catalpol	acute cerebral ischemia	To investigate the feasibility of catalpol intranasal administration and its protective effect on acute cerebral ischemia in rats via anti-oxidative and anti-apoptotic mechanisms.	Intranasal administration of catalpol to evaluate the nasal mucosal toxicity, brain targeting and pharmacokinetics. The protective effect of catalpol of intranasal administration on stroke-induced brain injury in rats and its mechanisms on	Catalpol intranasal administration was safe and feasible with no hemolysis, no bad effect on the maxillary ciliary movement of a bullfrog. Brain targeting index (DTI) of catalpol was greater than 1, which indicated that catalpol had good brain targeting after	Catalpol intranasal administration has good safety, stability and brain targeting. It can effectively protect the brain injury of the rat model of acute cerebral ischemia and provide the possibility of	(Wang et al., 2022)

				oxidative stress pathway Nrf2/HO-1 and apoptosis were also investigated using middle cerebral artery occlusion (MCAO).	intranasal administration	drug administration in the acute stage of cerebral ischemia.	
**4**	Dantrolene	multiple neurodegenerative diseases	To investigate if intranasal dantrolene administration produced better brain penetration than the regularly used oral method.	C57BL/6 mice (2-4 months old) were given a single dose of intranasal or oral dantrolene (5 mg/kg). Co-administration of P-gp/BCRP inhibitors, nimodipine, or elacridar, inhibited dantrolene clearance from the brain. At different time intervals after treatment, the concentration of dantrolene in the brain and plasma was determined.	Dantrolene concentrations in the brain were sustained for 180 minutes following intranasal delivery, but concentrations in the brain after oral administration decreased to zero after 120 minutes.	When compared to oral administration, intranasal administration of dantrolene is an effective way to boost its concentration and duration in the brain, with no noticeable negative effects on olfaction or motor performance.	(Shi et al., 2020)
**5**	Immunoglobulin G (IgG)	**CNS disorders**	Intranasal administration could be utilised to deliver antibodies as large as 150 kDa full-length IgG to the	By characterising antibody distribution, dose-response, and mechanisms of antibody transport to and within the brain after administering	Intranasal delivery also resulted in significantly higher [125I]-IgG concentrations in the CNS than systemic (intra-	It may be possible to attain therapeutic levels of IgG in the CNS, especially at greater	(Kumar et al., 2018)

			CNS, and antibody transport over the nasal epithelia and subsequent access to the perivascular regions of cerebral blood vessels could be identified and regulated for improved efficiency.	non-targeted radiolabeled or fluorescently labeled full-length immunoglobulin G (IgG) to normal adult female rats	arterial) delivery for doses producing similar endpoint blood concentrations.	intranasal doses, as well as determine the most likely cranial nerve and perivascular distribution channels used by antibodies to reach the brain from the nasal mucosae.	
**6**	Secretome	brain oxidative stress and neuroinflammation	To inhibit chronic ethanol and nicotine self-administration and relapse by the non-invasive intranasal administration of antioxidant and anti-inflammatory secretome generated by adipose tissue-derived activated mesenchymal stem cells.	Rats bred for their alcohol preference ingested alcohol chronically or were trained to self-administer nicotine. Secretome of human adipose tissue-derived activated mesenchymal stem cells was administered intranasally to animals, both (i) chronically consuming alcohol or nicotine and (ii) during a protracted deprivation before a drug re-access leading to relapse intake.	The intranasal administration of secretome derived from activated mesenchymal stem cells inhibited chronic self-administration of ethanol or nicotine by 85% and 75%, respectively. Secret ome administration fully abolished the oxidative stress induced by chronic ethanol or nicotine self-administration, shown by the normalization of the hippocampal	The non-invasive intranasal administration of secretome generated by human adipose tissue-derived activated mesenchymal stem cells markedly inhibits alcohol and nicotine self-administration, an effect mediated by the glutamate GLT-1 transporter.	(Quintanilla et al., 2019)

					oxidized/reduced glutathione ratio, and the neuroinflammation determined by astrocyte and microglial immunofluorescence.		
**7**	Rotenone	conditioned taste aversion memory	To investigate the effect of intranasal administration of rotenone on conditioned taste aversion memory in mice.	The neurotoxic effects of rotenone have been identified by using various routes of administration, and the most common route of administration is through a systemic administration including subcutaneous, intravenous, and intraperitoneal injections.	We found that the intranasal administration of rotenone impaired conditioned taste aversion memory to bitter taste in mice.	intranasal administration of rotenone decreases GABAergic synaptic transmission in layer V pyramidal neurons of the mouse insular cortex, the result of which leads to impairment of LTD and conditioned taste aversion memory	(Toyoda et al., 2020)
	hydrocarbon-stapled mimetic of relaxin-3	Depression and anxiety	Investigators proposed that a hydrocarbon-stapled mimetic of relaxin-3, when administered	A series of hydrocarbon-stapled relaxin-3 mimetics were designed and identified the most potent compound	An *i*,*i*+7 stapled relaxin-3 mimetic was synthesized that manifested a stabilized α-helical structure,	Preclinical findings demonstrate that targeting the relaxin-3/RXFP3	(Marwari et al., 2019)

			intranasally, might be uniquely applicable to the treatment of depression and anxiety.	using in vitro receptor binding and activation assays. Further, the effect of intranasal delivery of relaxin-3 and the lead stapled mimetic in rat models of anxiety and depression was assessed.	proteolytic resistance, and confirmed agonist activity in receptor binding and activation in vitro assays. The stapled peptide agonist enhanced food intake after intracerebral infusion in rats, confirming in vivo activity. Further, intranasal administration of the lead *i*,*i*+7 stapled peptide exerted anxiolytic and antidepressant-like activity in anxiety- and depression-related behaviour paradigms.	receptor system via intranasal delivery of an *i*,*i*+7 stapled relaxin-3 mimetic may represent an effective treatment approach for depression, anxiety, and related neuropsychiatric disorders.	
